# Cervicofacial cystic lymphangiomas in 17 childrens: A case series

**DOI:** 10.1016/j.amsu.2022.103835

**Published:** 2022-05-19

**Authors:** Omar Berrada, Mohamed Beghdad, Zineb El krimi, Youssef Oukessou, Sami Rouadi, Redalah LarbiAbada, Mohamed Roubal, Mohamed Mahtar

**Affiliations:** ENT Head and Neck Surgery Department, Ibn Rochd University Hospital, Faculty of Medicine and Pharmacy, Hassan II University, Casablanca, Morocco

**Keywords:** Cystic lymphangiomas, Surgery, Children

## Abstract

**Introduction:**

Cystic lymphangiomas are rare dysembrioplasias that occur mostly in children. Although benign, these tumors remain potentially life-threatening, due to the possible compression of the upper airway. The management of cystic lymphangiomas is still somewhat controversial, with surgery generally being the first-line treatment.

**Patients and methods:**

17 patients were included in this retrospective study, all aged less than 18 years old and treated for head and neck cystic lymphangiomas at our department between 2007 and 2017. All these patients had received surgical treatment alone. The relevant data were analyzed with SPSS software.

**Results:**

17 patients were included, with a sex ratio M/F of 1,4, and an average age of 4 years old. Complete resection of the tumor could only be completed in 12 patients. No postoperative complications were observed in our series. All the patients were followed for a minimum of 2 years after treatment.

**Conclusion:**

Cystic lymphangiomas are rare tumors of mysterious origins. The main symptom is swelling of the affected area. In our series, the results of the surgery were promising and consistent with results reported in the literature. A follow-up study with a larger population could be interesting, to further examine potential prognostic factors.

## Highlights

• Cystic lymphangiomas are rare dysembrioplasias.
• There are multidisciplinary approaches to the treatment of lymphatic malformations.
• Cystic hygroma posed a treatment challenge.

## 1 Introduction

Cystic lymphangiomas are rare benign dysembryoplasias of the lymphoganglionic system, responsible for a tumor syndrome by angio-lymphatic proliferation. Together with neurofibromas and hemangiomas, they belong to the group of hamartomas [1]. They are believed to arise from a blockage or arrest of normal growth of the primitive lymph channels during embryonic development [2].

Their anatomical localization is almost exclusively cervicofacial and their clinical revelation is generally very early in the neonatal period. The seriousness of these tumor formations in children is due, on the one hand, to may their evolutionary potential which may involve vital structures, such as the sympathetic chain, carotid sheath content, and branches of the hypoglossal, lingual, and facial nerves, and on the other hand, to the classic difficulty of their removal [3,4].

Cystic hygroma has posed a treatment challenge since it had been first described in 1843 by Wernher [5]. Although surgical extirpation is the preferred method of treatment [5].

Our work aimed to analyze the clinical and radiologic aspects of cystic lymphangiomas and determine the elements useful in making a correct diagnosis, as well as to specify possible prognostic factors for this condition and children, and evaluate the efficiency of surgical treatment. This work is reported in line with the PROCESS 2020 criteria [6].

## 2 Patients and Methods

This was a retrospective study led by the Otorhinolaryngology and Head and Neck Surgery department at the hospital on August 20th, 1953, in the university hospital of IBN ROCHD in Casablanca.

All the patients included were aged less than 18 years of age and were admitted to our department for cystic lymphangiomas.

We excluded from the study the patients who had t incomplete files and lost to follow-up.

The period of our study was 10 years, beginning in March 2007 and extending to March 2017.

All the patients received a complete clinical examination and appropriate imaging to establish the diagnosis and received surgical treatment alone.

The relevant data were collected from the patients’ clinical files and were analyzed using the SPSS software.

All the patients gave their written consent to be included in this study.

## 3 Results

### 3.1 Epidemiologic data

Seventeen patients were included in the study, of which 10 were male and 7 were female, with a sex ratio M/F of 1,4.

The mean age of our population was 3.99 years, with a standard deviation of 3.69 years. 35.29% of patients belonged to the 12–24 months age group. The youngest patient was 6 months old, and the oldest was 15 years old. None of the patients had a family history of similar affections.

### 3.2 Clinical findings

The patients presented to our facility for a medical consultation because of a lump or swelling localized in the neck area in 8 cases, in the facial region in 6 cases, and both the face and the neck in the remaining 3 cases. Only one patient presented with associated dysphagia. No other compressive signs, such as dyspnea or dysphonia, were reported.

**Fig. 1:**
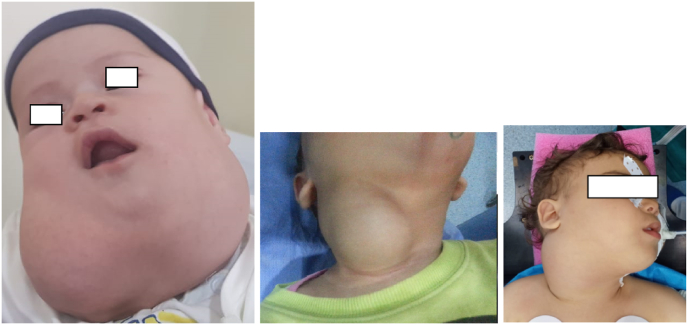
Massive lymphangioma in different children.

Clinical examination found a firm and polylobed mass, mobile about the superficial planes, and seemingly adherent to the deep tissues in all the patients (Fig. 1).

The mass extended to the floor of the mouth in 3 patients, and the parotid and sub-maxillary regions in 3 patients.

The average length of the mass was 7,22 cm (cm), with a standard deviation of 3,2 cm.

### 3.3 Complementary examinations

Out of our patients, 13 had an ultrasound first, which showed a mass made of macrocysts in 8 patients, and a microcystic mass in 5 patients.

**Fig. 2:**
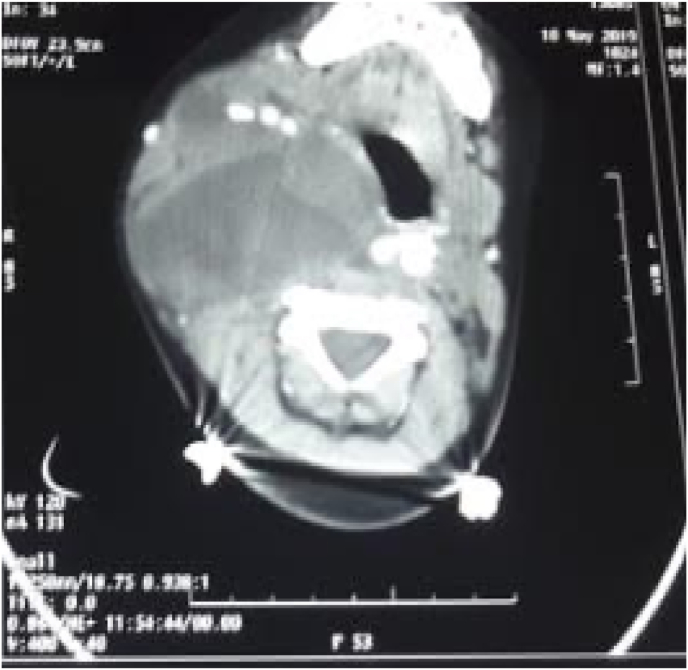
CT scan (C+)– axial section-hypodense mass compressing the aerodigestive tract.

All the patients underwent computed tomography (CT) scan of the neck; 10 patients benefited from a thoracic CT scan as well. The cervical CT scan was able to further approve the diagnosis of cystic lymphangioma, showing a polylobed cystic mass (Fig. 2).

The patient who presented with dysphagia benefited from an esophageal transit imaging with gastrograffine, which showed the compressive effect of the tumor on the cervical esophagus.

Endoscopic examination was not deemed necessary in any of the patients.

### 3.4 Treatment and evolution

**Fig. 3:**
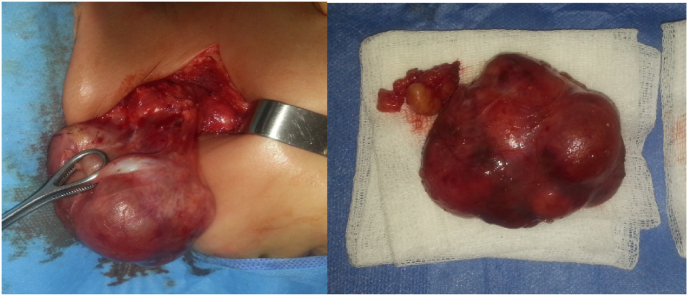
Preoperative tumor picture of a right-sided cervical mixed lymphangioma in a 3-year-old boy.

All the patients received surgical treatment performed by ENT Professor. Complete resection of the mass was possible only in 12 patients (Fig. 3).

The average length of hospitalization after surgery was 3 days. There were no immediate postoperative complications observed in our series.

14 patients were then seen and observed at our consultation for 3 years; the other 3 did not come after the first year. The first follow-up after surgery was done at 2 weeks. The patients were then checked every 3 months for the first year, and every 6 months for the remaining 2 years.

Complete remission was observed in 10 patients, with the total disappearance of the swelling, while 4 patients had partial remission and reported a significant improvement, but with the persistence of light swelling.

To this date, no death has been recorded in our series.

## 4 Discussion

Cystic lymphangiomas are rare benign tumors that can occur in any region of the body, but the cervicofacial region accounts for up to 75% of all cystic lymphangiomas [7,8]. They represent between 2.6 and 5% of benign cervical masses [9]. The condition incidence is estimated at one in 2000–6000 newborns. The symptoms are represented by pain and local swelling, especially during periods of regional infection and deformations of the anatomic structures [4]. We collected 17 cases of cystic lymphangiomas in children over 11 years, with a frequency of 1.54 cases per year. This rate is higher than that reported by Ozen et al. (17 cases in 20 years) [10] but lower than that reported by Triglia (three cases per year) [11] and Tekou (1.8 cases per year) [12].

The sex ratio varies in literature; in our series, there is a predominance of males (sex ratio: 1.4), and the same tendency was reported in several studies [11]. Other series report a female predominance, such as that of François [13].

Although of dysembryoplastic origin, the cyst is present at birth in only 50–60% of cases but is expressed in 90% of cases before the age of two years [13]. The age of discovery in our patients corresponds relatively to the data found in the literature, with a peak in frequency after the age of 2 years, and an average age of discovery at 3.99 years.

In the neck, cystic lymphangiomas are classically located in the posteroinferior triangle [14].

In most cases, the diagnosis of lymphangioma is made when a localized swelling is discovered. It is a soft and depressible mass of variable size (usually moderate), not very prominent. The skin covering the mass can be normal or lilac-pink, and is usually smooth, though some cases of hyperkeratotic skin or vesicular lesions have been reported [15,16].

Lymphangiomas are generally slow-growing, but can sometimes evolve rapidly, and in rare cases, the mass may decrease in size or even disappear completely before reappearing later [17]. They evolve in inflammatory flare-ups triggered by a nearby infection, and intra-lesional hemorrhages are frequent, leading to sudden increases in volume [18].

In our study, lymphangiomas were revealed by the appearance of a mass of variable size, poly-lobed, of soft consistency, progressively increasing in volume, limiting the movements of the neck, without any vascular-nerve involvement.

Complementary imaging is necessary to reinforce the diagnosis and assess the tumor's relation to the nearby structures and vessels, given an eventual surgical intervention.

Ultrasound is the first-line examination, to help specify the location and nature of the mass. The echo structure of cystic lymphangioma is typically similar to liquid or cystic lesions, organized in microcysts or macrocysts [19].

Computed tomography (CT) scan shows a hypodense, well-circumscribed mass without invasion of surrounding anatomical structures. MRI is complementary to CT to study the relationship of lymphangioma with the surrounding structures [20]. In our series, CT scanning was performed on all our patients.

Several classifications have been proposed to classify lymphatic malformations, based! on morphological aspects. A first classification divides lymphangiomas into microcystic, macrocytic and mixed [4].

Recently, another classification was added to this widely used classification, according to the microscopic aspect: lymphangioma simplex (lymphangioma circumscriptum), characterized by the presence of thin-walled lymphatic vessels; cavernous lymphangioma, characterized by dilated lymphatic vessels; cystic lymphangioma (CH), with huge, macroscopic lymphatic spaces; and benign acquired progressive lymphangioma, in which the lymphatic channel dissects the dense collagen bundles [21].

There are multidisciplinary approaches to the treatment of lymphatic malformations with the priority being the patient's quality of life. Surgery is no longer a primary modality of treatment, It may be important for patients with life-threatening problems, after sclerotherapy failure, and for those who have the microcystic disease. It appears to be less morbid and more effective when performed at tertiary centers with experienced lymphatic malformation surgeons [21]. In our study, all patients underwent an exclusive surgical treatment with a good evolution.

Surgical treatment carries risks.

Postoperative complications occur in 30% or more cases [22].

The frequency of Postoperative complications occurs in 30% or more cases such as FREY syndrome or CLAUDE BERNARD HORNER syndrome, airway obstruction, severe infections, or chylothorax.

In our study, the patients were treated by surgery alone with an estimated emission rate of 82%. In comparison with the literature, FLANAGAN reported a success rate of surgical treatment of 75%, with a follow-up of more than 5 years in 74% of patients [12]. HANCOCK reported a remission rate of 77.4% for all locations combined (52.5% of tumors were located in the head and neck) [22].

The same was true for ALQAHTANI which obtained a complete improvement in 77% of patients (34.9% of tumors of the head and neck) [23].

Current treatment options include non-operative modalities, sclerotherapy, radiofrequency ablation, and laser therapy. New therapies are emerging, including sildenafil, propranolol, sirolimus, and vascularized lymph node transfer [21]. In our study, no patient received sclerotherapy.

## 5 Conclusion

In our study population, no patient had an associated congenital anomaly or family history of lymphangioma.

The skin swellings were the major clinical symptom, while the visceral localizations were only found during complications.

The results of surgical treatment were consistent with the literature.

The study of prognostic factors suggested a favorable role for the macro cystic type. The microcystic type, the initial inflammatory symptomatology, and the anti-inflammatory treatment could favor the occurrence of relapses. The role of the age of discovery, the location, and the number of lesions could not be demonstrated.

We were not able to statistically confirm these hypotheses due to the small number of patients included (lack of statistical power and a high number of patients lost to follow-up). It would be very interesting to continue this study to be able to affirm our conclusions with certainty, because of their potential therapeutic impact.

## Funding

This research did not receive any specific grant(s) from funding agencies in the public, commercial, or not-for-profit sectors.

## Consent

Written informed consent was obtained from the patient for publication of this case report and accompanying images. A copy of the written consent is available for review by the Editor-in-Chief of this journal on request.

## Registration of research studies

This is a case report that does not require a research registry.

## Provenance and peer review

Not commissioned, externally peer-reviewed.

## Please state any conflicts of interest

The authors declare that they have no competing interests.

## Please state any sources of funding for your research

This research did not receive any specific grant(s) from funding agencies in the public, commercial, on non-for-profit sectors.

## Ethical approval

I certify that this kind of manuscript does not require ethical approval by the Ethical Committee of our institution.

## Consent

Written informed consent was obtained from the patients for publication of this cohort study and accompanying images. A copy of the written consent is available for review by the Editor-in-Chief of this journal on request.

## Author contributions

Omar Berrada: writing the paper, study concept.

Mohamed beghdad: writing the paper, study concept.

Zineb El krimi: acquisition of data.

Youssef Oukessou: study concep

Sami Rouadi: revising the article.

Redallah Larbi Abada: revising the article.

Mohamed Roubal: revising the article.

Mohamed Mahtar: final approval of the version to be submitted.

## Registration of research studies

This is a cohort study that does not require a research registry.

## Guarantor

Omar Berrada.

## Declaration of competing interest

The authors declare that they have no competing interests.
